# Microscopic Mechanisms of Solute Distribution Patterns Dominating Defect Evolution and Strengthening-Toughening in Iron-Based Solid Solutions

**DOI:** 10.3390/ma19143118

**Published:** 2026-07-21

**Authors:** Ning Dang, Huan Liu, Junfeng Cao, Jianjun Wang, Yiwen Xu, Lihong Han, Anqing Fu

**Affiliations:** 1 State Key Laboratory of Oil and Gas Equipment, CNPC Tubular Goods Research Institute, Xi’an 710077, China; 2 School of Mechanics and Transportation Engineering, Northwestern Polytechnical University, Xi’an 710072, China; liuhuan123@mail.nwpu.edu.cn; 3 PetroChina Changqing Oilfield Company, Qingyang 745000, China; 4 Oil Production Technology Research Institute, PetroChina Xinjiang Oilfield Company, Karamay 831399, China

**Keywords:** iron-based alloys, molecular dynamics, distribution patterns, solid solution strengthening, dislocation evolution, strengthening-toughening mechanism

## Abstract

To meet the stringent requirements for the synergy of ultra-high strength and exceptional toughness in drilling equipment for ultra-deep wells, this study employs molecular dynamics (MD) simulations to systematically investigate the intrinsic effects of spatial configurations (Cluster vs. Dispersion) of five transition metal elements (Co, Mo, Ni, Ti, and W) in an iron matrix on micro-defect evolution and mechanical performance. The simulation systems, each containing approximately 54,000 atoms, were initially relaxed under an isothermal-isobaric (NPT) ensemble at 300 K for 50 ps to ensure thermodynamic equilibrium, and were subsequently deformed under uniaxial tensile loading at a strain rate of 5 × 10^9^ s^−1^. The results demonstrate that the distribution pattern of solute atoms influences the mechanical response. The cluster configuration generally weakens the matrix strength, a behavior indicative of stress localization and dislocation pile-up at the interfaces; in contrast, the dispersed configuration is associated with a more thermodynamically stable state and enhanced ultimate load-bearing capacity. Specifically, the Fe-Mo dispersed system reaches an ultimate tensile strength (UTS) of 28.32 GPa under the studied conditions, which exceeds the pure Fe benchmark of 24.90 GPa—an enhancement that points toward the formation of denser local atomic ordering and extensive dislocation network hardening. Meanwhile, the Fe-W dispersed system exhibits sustained dislocation activity up to large strains, suggesting that enhanced cross-slip contributing to its relatively high ultimate tensile strain and toughness. Based on these atomistic insights, a “Mo-W composite dispersed” micro-configuration design strategy is proposed as a conceptual direction for further exploration.

## 1. Introduction

Ultra-deep drilling at the 10,000 m scale is a core endeavor in deep-earth energy exploration. In this extreme environment, rock-breaking tools must withstand hydrostatic pressures on the order of hundreds of megapascals and severe high-frequency impacts from extremely hard rock layers [1,2]. Such “high-pressure-impact” conditions demand matrix materials that break the traditional strength-ductility trade-off to achieve synergy of ultra-high strength and exceptional toughness [3]. Iron-based alloys have become the preferred candidates due to their superior load-bearing potential; thus, elucidating the intrinsic strengthening-toughening mechanisms of alloying elements at the atomic scale is crucial for advancing the performance limits of deep-earth equipment [4]. Recent macro-experimental investigations on advanced drilling tool steels, including ultra-high-strength low-alloy steels and maraging steels, have explicitly highlighted that localized solute segregation and atomic-scale clustering severely deteriorate toughness, shift the ductile-to-brittle transition behavior, and trigger premature fatigue crack initiation under high-frequency cyclic loading [5,6,7]. Despite these experimental insights, capturing the transient, sub-nanometer interaction between evolving defect networks and specific local solute topologies via in situ experimental techniques remains fundamentally constrained. To strictly decouple thermal softening from topological strengthening mechanisms, this foundational study sets the baseline thermodynamic condition at 300 K.

The introduction of transition metal elements is a widely recognized approach to modulating the micro-defect dynamics and mechanical response of iron-based alloys. This study systematically selects five typical transition metals—Co, Mo, Ni, Ti, and W—as doping solutes. From the standpoint of tribology and surface fatigue, this selection is highly deliberate: refractory elements like W, Mo, and Ti impart exceptional wear resistance under harsh friction, whereas Co and Ni enhance matrix toughness and cyclic strain-hardening capacity, fundamentally delaying fatigue failure [8,9,10,11]. Beyond their engineering merits, these five elements span different periods and groups, possessing significantly distinct atomic radii and outer d-orbital electronic configurations [12]. Large-sized refractory metals such as Mo and W readily induce severe lattice distortion in BCC iron, constructing high-energy barriers that impede dislocation slip [13,14]; conversely, Ni and Co, through their role in modulating stacking fault energy, are thought to facilitate plastic deformation mechanisms such as localized cross-slip [15]. A systematic comparison of these elements therefore comprehensively covers the complex microscopic strengthening–toughening landscape.

In recent years, molecular dynamics (MD) simulations have been widely used to reveal nanomechanical behaviors of alloys. Recent methodological advancements have significantly expanded the spatiotemporal capabilities and physical accuracy of atomistic investigations in BCC iron systems. For instance, the development of hybrid off-lattice kinetic Monte Carlo (MLKMC) algorithms, which couple kinetic Activation–Relaxation Techniques (k-ART) with rigid-lattice atomistic kinetic Monte Carlo (AKMC), has successfully captured the long-term dynamic diffusion of solute atoms and the formation kinetics of Cottrell atmospheres around screw dislocations [16]. Furthermore, deep learning-assisted hybrid Monte Carlo simulations have demonstrated that chemical short-range ordering (SRO) configurations under extreme thermodynamic conditions serve as a governing mechanism stabilizing the cubic BCC lattice against phase transitions [17]. To circumvent the empirical limitations of conventional interatomic potentials in complex segregation zones, machine learning force fields (MLFF) trained on ab initio data have also been deployed to quantify the segregation energies and local coordination evolutions of alloying elements (such as P and Si) at symmetric tilt grain boundaries in BCC iron [18]. However, existing MD studies are largely polarized: they either focus on ideal high-entropy alloys or concentrated solid solutions to explore solid solution strengthening under absolute dispersion [13,19], or independently investigate specific nanoprecipitates (e.g., Cu-rich clusters, carbides) to study interfacial instability and dislocation bypassing mechanisms [20,21]. In practical material fabrication, constrained by thermodynamics and kinetics, the spatial distribution of solute atoms exists along a dynamic spectrum between “dispersion” and “cluster.” Few studies have conducted rigorous controlled comparisons of the intrinsic mechanical differences between these two distribution patterns for the same solute element. In the context of material fatigue, we propose a working hypothesis that the spatial segregation of elements may play a critical role in early crack initiation: we postulate that rigid solute clusters may act as intense localized pinning centers during cyclic or impact loading, potentially triggering severe dislocation pile-ups and stress concentrations that could nucleate micro-voids and fatigue cracks. Conversely, a globally dispersed configuration might homogenize dislocation glide and alleviate localized stress, thereby fundamentally delaying crack initiation.

To test this hypothesis, this study employs MD simulations using a pure BCC iron matrix to construct two atomic configurations—a “central cluster” and a “globally dispersed” configuration—doped with equal volume fractions of the five solute elements. Under extremely high strain-rate tensile loading, we combine atomic potential energy tracing, radial distribution function (RDF) structural evolution, and Dislocation Extraction Algorithm (DXA) techniques to analyze the dominant role of spatial distribution patterns on mechanical response. Unlike previous atomistic studies that emphasize either absolute solid solution strengthening or isolated precipitate bypass mechanisms, our comparative investigation is designed to evaluate whether dispersed topologies promote stable multi-slip dislocation networks (resembling recent findings on short-range ordering behaviors) and whether localized clusters act as severe stress concentrators leading to premature failure. A primary objective of this work is to decouple the topological dimension (spatial configuration) from the chemical dimension (solute concentration), unveiling how the spatial arrangement of solutes influences the trade-off between strength and maximum uniform strain. This provides a novel microstructural design strategy—manipulating spatial clustering rather than merely altering chemical compositions. It should be noted that, to isolate the independent topological effects of solute distribution, this study deliberately adopts a simplified model system and does not incorporate the full microstructural complexity of engineering steels (e.g., carbon, carbides, grain boundaries, multiphase constituents, and solute segregation). These factors, along with elevated-temperature conditions relevant to deep drilling, represent important directions for subsequent multiscale investigations.

## 2. Methods

### 2.1. Simulation System Construction

**Figure 1:**
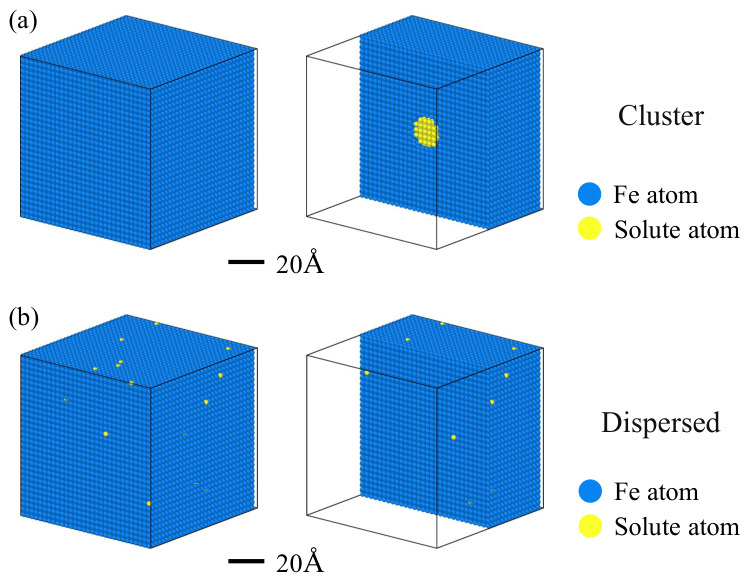
Schematic illustration of the solute distribution configurations in the pure iron matrix: (**a**) Cluster, (**b**) Dispersed.

All molecular dynamics (MD) simulations in this study were conducted using the open-source Large-scale Atomic/Molecular Massively Parallel Simulator (LAMMPS, version 22 July 2025). To systematically evaluate the impact of solute distribution patterns on the mechanical behaviors of iron-based alloys, this section utilizes a body-centered cubic (BCC) pure iron matrix and five typical transition metal elements (Co, Mo, Ni, Ti, and W) as doping solutes. Two extreme spatial distribution patterns—the “central cluster” and the “globally dispersed” configurations—were constructed for the atomic models, as shown in Figure 1. The simulation cell dimensions were set to 85.992 × 85.992 × 85.992 Å^3^, containing approximately 54,000 atoms. To eliminate artificial free surface effects and accurately mimic the macroscopic bulk response of the materials, strictly periodic boundary conditions (PBCs) were applied in all three spatial directions (x, y, and z) throughout both the thermodynamic relaxation and the tensile loading phases. Crucially, considering that the five transition metal solutes possess significantly distinct atomic radii, a fixed volume fraction—calculated at approximately 0.042 vol.%—was rigorously employed as the global baseline metric rather than a fixed atomic percentage (at.%).

- Cluster model: A spherical cluster of solute atoms with a fixed diameter of 8 Å (corresponding to the 0.042 vol.% macroscopic concentration) is constructed at the center of the simulation cell. This specific sub-nanometer dimension was selected to physically represent the early-stage formation of incipient solute domains, while geometrically ensuring that its localized strain field fully dissipates within the ~86 Å cell to prevent artificial self-interactions across the PBCs.
- Dispersed model: Solute atoms randomly replace an equal number of Fe atoms as present in the respective element’s cluster model throughout the entire domain. Specifically, this uniform solid solution was generated utilizing the native stochastic substitution algorithm built into LAMMPS (via the set command with a specified random seed). This approach guarantees an ideal stochastic distribution with maximum configurational entropy while intentionally avoiding Monte Carlo (MC) methods that might spontaneously induce artificial short-range ordering (SRO). This ensures that for each specific solute element, the total number of solute atoms remains strictly identical between its clustered and dispersed configurations, thereby completely eliminating the interference of concentration variations and exclusively isolating the topological effect of spatial distribution.

Immediately following the geometric construction, a strictly defined Conjugate Gradient (CG) energy minimization was performed on all models. This crucial static step mechanically alleviates unphysical initial local stresses and artificial atomic overlaps induced by the atomic size mismatch between the BCC Fe matrix and the solutes, preparing the systems for subsequent thermodynamic dynamic equilibration.

### 2.2. Simulation Parameters and Loading Protocols

In molecular dynamics simulations of multicomponent alloys, the selection of the interatomic potential directly determines the accuracy of lattice distortion, defect evolution, and mechanical responses. This study employs the Embedded-Atom Method (EAM) alloy potential developed by Zhou X. W. et al. (2004) [22] to describe the interatomic interactions within the system.

The total energy expression $E_{total}$ for this potential is given by:

(1) $$E_{total}=\frac{1}{2}\sum_{i,j(i\neq j)}\phi_{ij}(r_{ij})+\sum_{i}F_{i}(\rho_{i})$$

In the above expression, $\phi_{ij}$ is the pair potential function between atoms i and j, $r_{ij}$ is the interatomic distance, and $F_{i}$ represents the embedding energy required to place atom i into a local electron density environment $\rho_{i}$.

The core advantage of selecting this potential lies in its extensive library, which covers 16 metallic elements including Fe, Co, Mo, Ni, Ti, and W. The cross-interactions between heterogeneous atoms are fitted and calibrated through a set of unified, universal mixing rules, which were originally validated against an extensive database of experimental measurements and first-principles density functional theory (DFT) calculations. This not only ensures an accurate description of pure-component properties (such as lattice constants, elastic moduli, and sublimation energies) but also guarantees, to the greatest extent possible, a fair and systematic comparison of the strain field differences and solid solution strengthening effects induced by these five distinct solute elements in the iron matrix under the same physical benchmark, even during severe deformation stages.

The simulation system utilizes the aforementioned potential and is relaxed for 50 ps within an isothermal-isobaric (NPT) ensemble at 300 K to ensure the system reaches thermodynamic equilibrium. Subsequently, uniaxial tensile loading is performed along the [100] direction (or x-direction) of the simulation cell. During the dynamic loading process, the system temperature is strictly maintained at 300 K utilizing a Nosé–Hoover thermostat, and the lateral pressures (y and z directions) are maintained at zero via a barostat algorithm (NPT ensemble) to simulate a uniaxial stress state. The loading rate is set to $5\times 10^{10}\;s^{-1}$. It should be explicitly acknowledged that while this ultra-high strain rate is several orders of magnitude higher than the actual macroscopic impact loads encountered in deep-earth drilling (typically 10^2^~10^4^ s^−1^), it is an inherent methodological requirement dictated by the femtosecond spatio-temporal scale limitations of molecular dynamics. In the context of this study, rather than directly replicating macroscopic field conditions, this accelerated loading acts as a theoretical “stress test”. It effectively isolates the instantaneous nucleation of defects and amplifies the topological effects of solute spatial distributions on dislocation kinetics under extreme dynamic shock limits. Consequently, it provides a fundamental upper-bound reference for the microstructural design of impact-resistant drilling materials. Throughout the simulation, the time step is set to 1 fs, and stress–strain data are recorded in real-time using the Virial statistical method.

### 2.3. Microanalysis Toolkit

To elucidate the strengthening-toughening mechanisms at the atomic scale, the following characterization tools are introduced in this study:

- RDF: The radial distribution function g(r) is utilized to quantify the degree of lattice distortion induced by solute atoms. By analyzing the changes in the peak intensity and the full width at half maximum (FWHM) of the first peak of g(r), the extent of lattice symmetry disruption caused by different distribution patterns is evaluated.
- DXA: The DXA is employed to identify defect evolution during deformation in real-time. Emphasis is placed on observing the density variations of 1/2<111> and <100> dislocations to explain the toughening effects and strengthening mechanisms of the elements.
- Average Potential Energy per Atom: The average potential energy per atom for each configuration is obtained by extracting and normalizing the total potential energy of the system at equilibrium. By comparing the energy differences between the dispersed and cluster states, the thermodynamic stability and pre-deformation energy storage of different distribution patterns are evaluated.
- Strain Distribution Maps: Local atomic displacement gradients are calculated using OVITO software (version 3.14.0) to generate Von Mises strain distribution maps. The strain distribution at the ultimate tensile strength (UTS) point is specifically observed to analyze the influence of cluster interfaces and dispersed lattices on stress concentration and micro-crack initiation.

## 3. Results and Discussion

### 3.1. Dominant Role of Solute Elements and Distribution Patterns on the Intrinsic Mechanical Response

**Figure 2:**
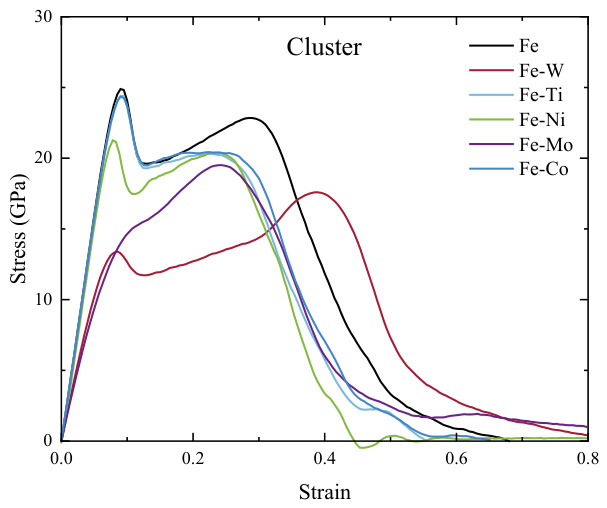
Uniaxial tensile stress–strain curves of pure iron and cluster-doped systems.

**Figure 3:**
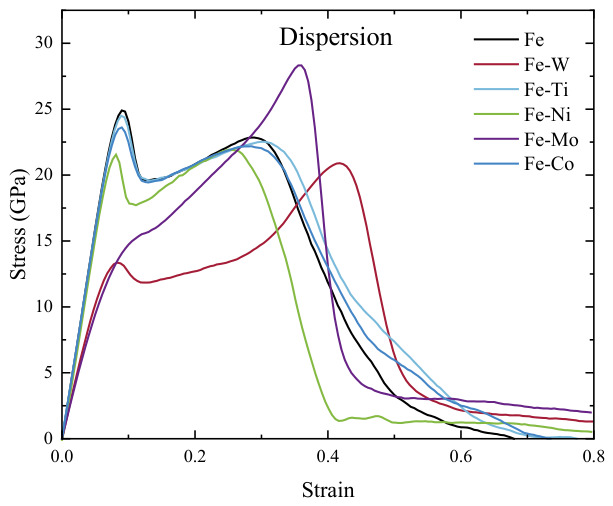
Uniaxial tensile stress–strain curves of pure iron and dispersed-doped systems.

Figure 2 and Figure 3 illustrate the uniaxial tensile stress–strain response curves for the five solute elements in both the cluster and dispersed configurations, respectively. The corresponding UTS and ultimate strain values are summarized in Table 1. In all tested systems, a significant linear elastic deformation is observed during the initial stage of tension, followed by entry into the plastic yielding stage, eventually leading to varying degrees of structural instability and stress drops.

The foundational reference to be established first is that the UTS of the pure Fe matrix reaches 24.90 GPa. It is imperative to note that the absolute strength values obtained in this study are conditioned by the intrinsic spatio-temporal limitations of MD simulations, specifically the ultra-high uniaxial strain rate of $5\times 10^{10}\;s^{-1}$. Under such extreme loading, thermal activation mechanisms (e.g., dislocation nucleation) are severely suppressed due to the insufficient timescale for thermal fluctuations. Consequently, the system is forced to deform through the direct stretching of metallic bonds, leading to a UTS that approaches the ideal theoretical strength of BCC iron. While these values align with previous first-principles (DFT) calculations and high-strain-rate MD simulations [23,24], they should be interpreted as theoretical upper bounds rather than directly transferable engineering strength values for real-world drilling tools.

Figure 2 visually reflects the weakening effect of “cluster” doping on the system strength. Compared with the pure Fe benchmark, when solute atoms are locally aggregated in the form of clusters, the ultimate stress of most systems exhibits varying degrees of decline. Among them, the Ti-cluster doped system retains the relatively highest strength within the group (24.40 GPa), while the W-cluster system shows the most significant reduction in UTS, dropping to 17.59 GPa. However, regarding the strain characteristics, the W-cluster system exhibits an extremely gentle declining segment after crossing the stress peak, with its ultimate strain reaching 0.3846, demonstrating remarkable ductility. It should be noted that in this study, the ‘maximum uniform strain’ is quantitatively defined as the critical strain corresponding to the UTS on the stress–strain curve. This value marks the physical transition point where the system’s uniform plastic deformation limit is exhausted, followed by the onset of strain localization and structural collapse. From the perspective of continuum mechanics and microscopic fracture physics, this general decline in strength may be related to a “physical interface effect” between the clusters and the matrix. The high density of heterogeneous atoms introduces substantial lattice mismatch at the cluster boundaries. Under extremely high applied loads, such discontinuous interfaces are plausible sites for local stress concentration, which could contribute to the observed premature yielding and reduced strength [25].

Figure 3, by contrast, demonstrates a markedly different trend: the dispersed configuration is associated with enhanced strength in most of the iron-based alloy systems studied. In the dispersed distribution mode, the UTS of all doped systems—with the exception of the Co element—fully surpasses that of their cluster-state counterparts. The most remarkable phenomenon occurs in the Fe-Mo dispersed system, where the entire tensile curve is significantly elevated, and the UTS surges to 28.32 GPa. This value not only ranks first among the five doping elements but also exceeds the UTS obtained for the pure iron matrix under the same simulation conditions. Furthermore, the W element further releases its toughening potential in the dispersed state, with its ultimate strain expanding to 0.4180, the largest within this group.

This inversion of the macroscopic mechanical response with spatial configuration suggests that, under the extreme loading conditions studied here, the dispersed solute distribution facilitates a distinct strengthening mechanism compared to the clustered arrangement. The dispersed distribution of solute atoms increases the effective elastic interaction volume between the solute and matrix atoms, thereby contributing to a globally distributed solid-solution strengthening effect [12]. In contrast to the locally concentrated mismatch at cluster interfaces, the dispersed configuration promotes a more uniform distribution of the applied work throughout the lattice, which could explain the observed improvement in strength. This fundamental reversal in mechanical behavior indicates that the underlying energy states and microstructural stability within the system are likely to have undergone substantial changes, motivating the energy analysis presented in the following section.

### 3.2. Characteristics of System Energy Storage and Microstructural Distortion

To elucidate the dominant role of distribution patterns on the macroscopic mechanical response from the perspective of underlying physical mechanisms, this section further traces the origin of the initial thermodynamic states and micro-static structural evolution of the systems.

**Figure 4:**
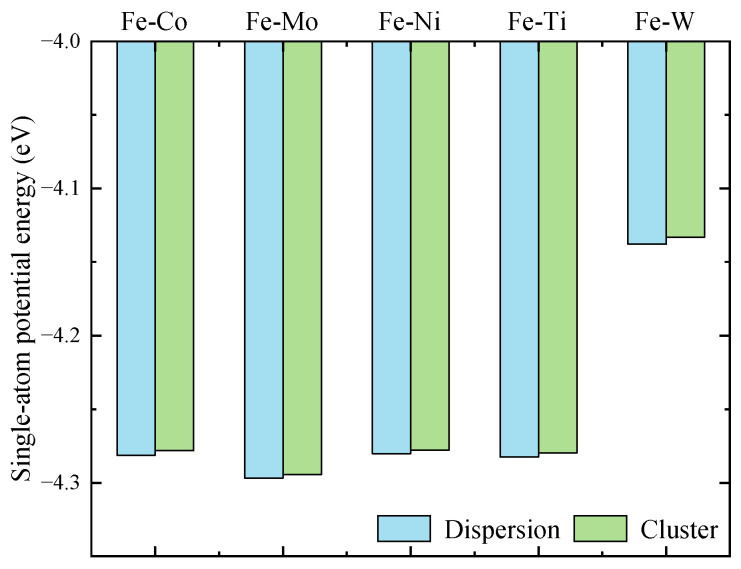
Comparison of the average potential energy per atom for different doped systems under various distribution patterns.

Figure 4 compares the average potential energy per atom of different doped systems before tension (at the relaxation equilibrium state). The data indicate that the average potential energy per atom for all tested systems is negative. A consistent feature across all doping combinations is that the absolute value of the average potential energy per atom for the dispersed configuration is explicitly greater than that of its corresponding central cluster configuration. This implies that the actual potential energy of the dispersed state is more negative, and the system as a whole resides in a stable state with lower energy. Among them, the Fe-Mo dispersed system reaches the minimum extreme value of potential energy per atom for this group. In sharp contrast, the Fe-W system exhibits a peculiar energy background: the absolute value of the potential energy per atom for Fe-W remains the smallest (i.e., the potential energy is at the highest level) in both the cluster and dispersed states.

This difference in energy states provides a thermodynamic rationale for the inversion of the macroscopic strengthening-toughening behavior. In the cluster configuration, the high-density aggregation of heterogeneous solute atoms forms distinct “phase interfaces.” These interfaces tend to introduce high lattice mismatch energy and interfacial excess energy, leading to an elevation of the overall potential energy, which may serve as a latent trigger point for structural instability [26]. Conversely, the dispersed configuration substantially reduces these high-energy interfaces; the solute atoms form an extensive and energetically favorable bonding network with the surrounding Fe atoms, allowing the entire system to occupy a more thermodynamically stable state. When subjected to ultra-high strain rate tension, a large amount of external mechanical work is required to disrupt this stable bonding network. This energy requirement could partly explain the notably high UTS observed in the Fe-Mo dispersed system. Meanwhile, the elevated baseline potential energy of the Fe-W system indicates that its lattice resides in a relatively flexible high-energy metastable state, granting the structure higher degrees of freedom for configurational reorganization and error tolerance. This provides a thermodynamic perspective that may contribute to its enhanced ductility [27]. These thermodynamic considerations, however, do not act in isolation; they operate in concert with the local elastic strain fields from atomic-size mismatch (Section 3.2, RDF analysis), the elastic modulus mismatch effects at interfaces (Section 3.3), and the dislocation-level mechanisms discussed in Section 3.4.

**Figure 5:**
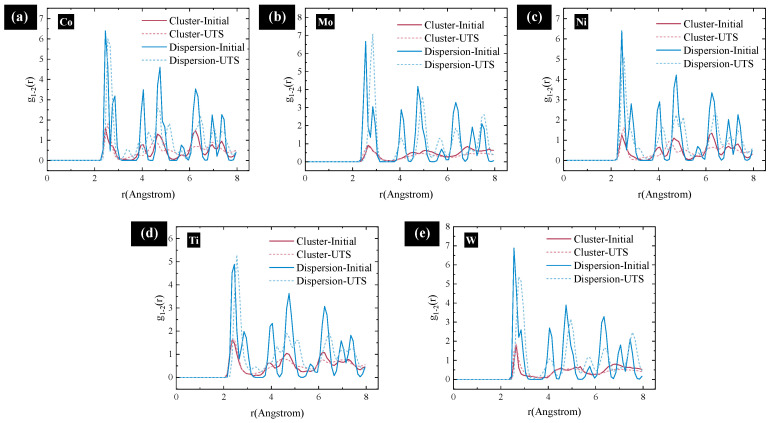
Radial distribution functions (RDF) of various systems at the initial state and ultimate tensile strength (UTS): (**a**–**e**) represent the doped systems corresponding to Co, Mo, Ni, Ti, and W elements, respectively.

To further explore the mapping of these thermodynamic characteristics onto the microscopic crystal structure, Figure 5 extracts the RDF between Fe atoms and solute atoms within a range of 0 to 8 Å for the solute-doped systems, comparing the evolution of neighbor coordination in each system at both the initial equilibrium state and the UTS state. As a one-dimensional, volume-averaged structural descriptor, the RDF captures changes in the average local coordination environment but does not directly resolve the full three-dimensional atomic configuration; accordingly, the structural interpretations offered below represent plausible inferences drawn from the RDF data rather than unique structural determinations.

Since this section extracts the g(r) curves for Fe–solute atom pairs, the intensity of the first-nearest-neighbor peak directly reflects the local enrichment of Fe atoms around the solute atoms. By observing the initial state and the UTS state, a consistent trend can be identified: whether before loading or at the loading limit, the first-nearest-neighbor peak intensity for the dispersed-doped state of all five elements is explicitly higher than that of the corresponding cluster-doped state. From the perspective of physical metallurgy, this is because, in the cluster configuration, solute atoms primarily undergo segregation and bonding with the same species of heterogeneous atoms, resulting in a limited effective coordination number for Fe–solute pairs. Conversely, the dispersed configuration yields a substantially larger interaction volume between heterogeneous atoms, which points towards a potential increase in the degree of local chemical short-range ordering between the solute atoms and the Fe matrix [28]. This denser Fe–solute coordination shell correlates with the more thermodynamically stable state described above. We note, however, that the RDF alone cannot distinguish between a genuine chemically ordered SRO configuration and a purely geometric densification arising from the elimination of solute-solute nearest neighbors in the dispersed arrangement; both scenarios would produce an increased Fe–solute first-nearest-neighbor peak.

When the loading reaches the UTS state, the applied extreme tensile stress induces severe lattice deformation. Compared with their respective initial states, the first-nearest-neighbor peak positions of all models shift toward the larger spacing direction (to the right), which visually reflects the forced stretching of interatomic distances and the lattice expansion effect caused by extreme stress.

However, at the UTS state, the evolution of the first-nearest-neighbor peak intensity for each system exhibits a highly physically significant differentiation, revealing differences in the microscopic mechanisms of different systems in response to extreme loads:

(1) Lattice distortion and bond dissociation (peak intensity decrease): In the dispersed-doped Co, Ni, and W systems, as well as the cluster-doped Mo system, the first-nearest-neighbor peak intensity decreases significantly. This indicates that under extreme tensile strain, the local Fe–solute coordination structures in these systems are severely disrupted, undergoing intense static and dynamic lattice distortions [29]. Especially for the high-toughness Fe-W dispersed system, the observed disintegration of its local coordination structure and the loss of neighbor correlation suggest a deformation mechanism in which plastic work is dissipated through the breaking and reorganization of local bonds, which may contribute to its relatively high macroscopic fracture strain.
(2) Peak intensity remains stable or increases: Distinctly different from the aforementioned systems, the first-nearest-neighbor peaks of the remaining systems (particularly the Fe-Mo dispersed system, which reaches an ultimate tensile strength of 28.32 GPa under the studied conditions) show no obvious weakening at the UTS state and even exhibit an increase. This is the directly observed RDF evidence. The local atomic ordering between these dispersed solute atoms and the Fe matrix appears to possess notable resistance to tensile deformation. The observed increase in peak intensity suggests that, rather than being pulled apart under high tensile force, these local structures undergo further atomic densification. These persistent locally ordered regions may function as stable atomic-scale reinforcing sites within the matrix. One possible mechanism for this behavior is that when dislocation lines encounter these strongly bonded regions, additional work is required to disrupt them—analogous to the resistance expected from localized obstacles in classical Orowan-type descriptions [30]. However, we must acknowledge that alternative explanations may also exist. For instance, the localized peak densification might arise from complex multi-axial stress states generated during extreme non-equilibrium deformation, rather than solely indicating the presence of strengthening obstacles. While RDF analysis alone cannot provide an unambiguous structural determination, the observed peak evolution offers indirect evidence suggesting that the stability of local atomic ordering correlates with the strengthening observed in specific dispersed-doped systems (such as Mo).

### 3.3. Strain Localization and Failure Precursors Under Ultimate Bearing Capacity

**Figure 6:**
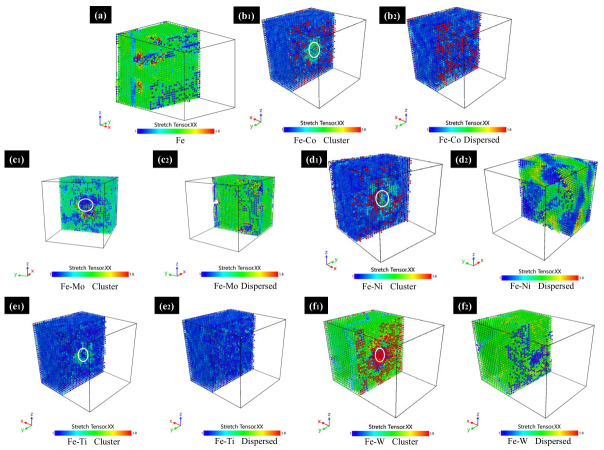
Local atomic strain distribution of pure iron and various doped systems under ultimate tensile stress: (**a**) Pure iron system, (**b_1_**–**f_1_**) Cluster-doped systems corresponding to Co, Mo, Ni, Ti, and W elements, (**b_2_**–**f_2_**) Dispersed-doped systems corresponding to Co, Mo, Ni, Ti, and W elements. White circles indicate the regions of the central solute clusters.

To visually reveal the microscopic deformation behavior prior to the macroscopic stress drop, Figure 6 extracts the local atomic equivalent strain distribution maps (Von Mises strain) for the pure Fe and various doped systems at the UTS state. The color gradient in the maps (from blue to red) characterizes the intensity of the local deformation at the atomic level.

Comparing the different distribution patterns reveals distinctly different strain localization morphologies. In the central cluster configuration systems, the high-strain regions (collections of red and yellow atoms) exhibit extreme localization. The extreme lattice deformation is concentrated almost entirely at the physical interface between the central solute cluster and the Fe matrix, while the matrix regions distant from the cluster maintain a large area of low strain (blue regions). In sharp contrast, in the dispersed configuration (especially the Fe-Mo dispersed system, which exhibits ultra-high UTS), the high-strain regions are no longer confined to a single core location. The red and yellow high-strain atoms are scattered at multiple points throughout the simulation cell, even interleaving into a network-like morphology that spreads extensively within the entire supercell matrix.

This morphological differentiation of strain maps at the UTS critical point indicates that the solute spatial configuration influences the microscopic deformation patterns preceding failure. From the perspective of microscopic solid mechanics and fracture physics, the extreme strain localization observed in the cluster configuration closely resembles the patterns that typically precede material failure and micro-crack nucleation. Due to the significant mismatch in lattice constants and elastic moduli between the cluster center and the Fe matrix, this semi-coherent or incoherent interface cannot effectively transfer the load outward under the ultra-high tensile stress during the transition from elastic to plastic deformation, resulting in severe stress concentration. The premature yielding observed at such interfaces may act as a limiting factor for the system, restricting the full activation of the load-bearing potential of the surrounding matrix. This interpretation offers a physical rationale for the generally lower UTS observed for cluster systems in Figure 2.

Conversely, the strain maps of the dispersed configuration are suggestive of a more globally distributed deformation pattern. Combined with the structural analysis in Section 3.2, the globally distributed lattice distortion and locally ordered structures associated with solute dispersion may create numerous small stress-perturbation fields within the matrix. When stretched to the ultimate state, these distributed internal distortions could facilitate a more uniform sharing of the applied mechanical work throughout the simulation cell. The absence of a single, continuous phase interface may contribute to the ability of the dispersed system to sustain higher external loads before the onset of localized failure [31].

These micro-strain distribution characteristics suggest a potential design principle for materials subjected to extreme loading conditions. The dispersed configuration, by avoiding the pronounced strain localization observed at cluster interfaces, may offer a strategy for distributing applied deformation more uniformly throughout the microstructure. We emphasize, however, that these atomistic observations are obtained from a simplified single-solute model system under a single loading condition. Whether such strain-delocalization mechanisms can be realized in the complex microstructures of engineering steels under realistic service conditions remains an open question for future investigation.

### 3.4. Dynamic Defect Evolution and Microscopic Plastic Deformation Mechanisms

**Figure 7:**
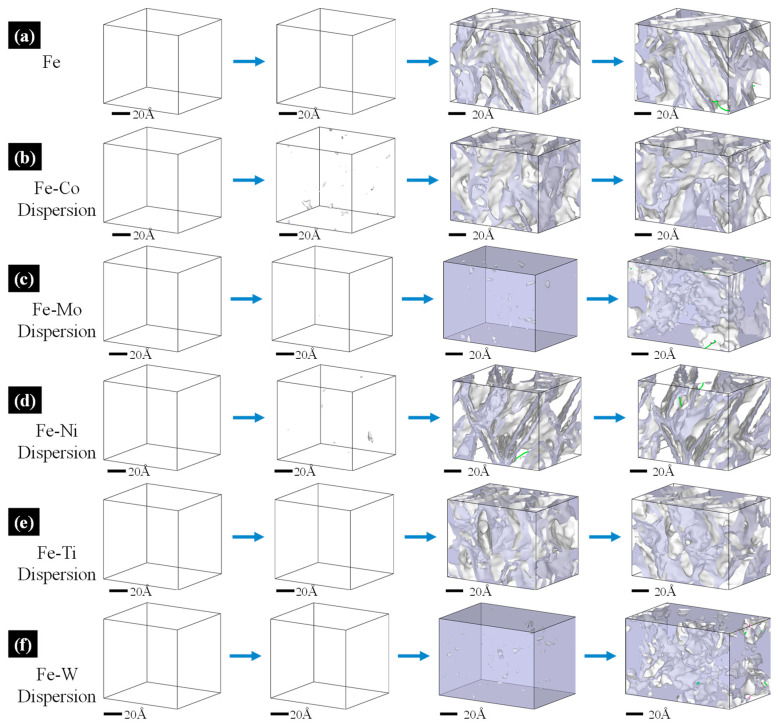
Microscopic dislocation evolution of pure iron and dispersed–doped systems at typical tensile stages (initial yielding stage, first peak, plastic flow/secondary hardening stage, and necking and failure initiation): (**a**) Pure iron system, (**b**–**f**) Dispersed-doped systems corresponding to Co, Mo, Ni, Ti, and W elements.

**Figure 8:**
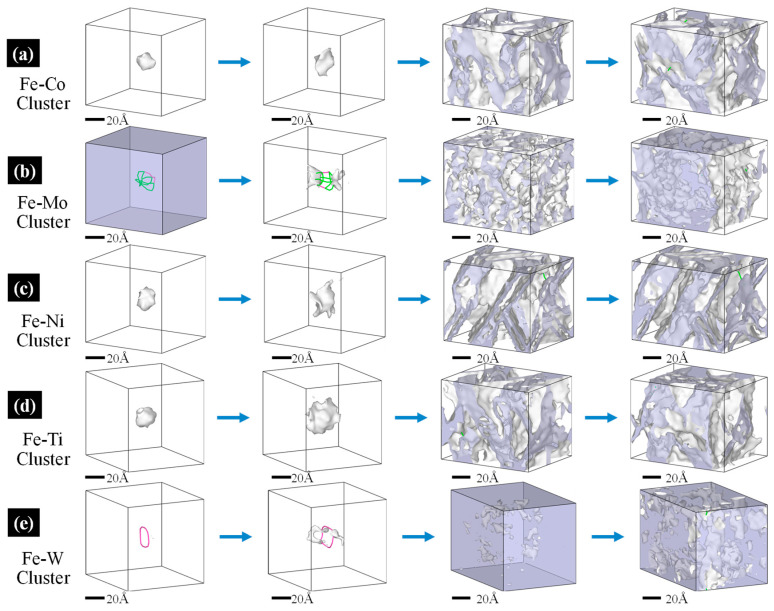
Microscopic dislocation evolution of cluster-doped systems at typical tensile stages (initial yielding stage, first peak, plastic flow/secondary hardening stage, and necking and failure initiation): (**a**–**e**) Cluster-doped systems corresponding to Co, Mo, Ni, Ti, and W elements. In the visualizations, green and pink lines denote 1/2<111> and <100> perfect dislocations, respectively.

The macroscopic mechanical response and strain localization behavior are essentially determined by the mechanisms of microscopic dislocation nucleation, multiplication, and motion. To visually reveal this micro-kinetic process, Figure 7 and Figure 8 utilize the DXA to demonstrate the 3D defect configuration evolution characteristics of the dispersed-doped and cluster-doped systems, respectively, during key tensile stages. It should be noted that DXA provides a static, dislocation-line-based representation of the defect structure; it does not directly resolve the dynamic processes—such as cross-slip, junction formation and dissolution, or dislocation bypassing—that are inferred from the observed configurations. The mechanistic interpretations offered in this section are therefore presented as hypotheses grounded in the observed defect morphologies and established dislocation theory, rather than as direct confirmations of specific dynamic events.

Comparing the first two stages at minimal strain reveals fundamental differences in the dislocation initiation mechanisms. In the cluster-doped systems shown in Figure 8, the deformation exhibits highly localized characteristics. Especially in the Mo- and W-cluster systems, green mobile dislocations (1/2<111> slip dislocations) and pink dislocation lines (<100> slip dislocations) can be clearly observed directly surrounding and adhering to the interfaces of the central clusters. This observation suggests that the phase interfaces between the clusters and the iron matrix act as preferential nucleation sources for dislocations, likely due to the substantial lattice distortion and stress concentration in these regions. In contrast, for the dispersed configurations in Figure 7 at the same stage, only sporadic defects are seen nucleating randomly throughout the cell. This dispersed nucleation pattern aligns with the picture proposed by classical solid-solution strengthening theory, in which dispersed solute atoms increase the lattice friction for dislocation slip through local elastic strain fields [12]. These distributed solute atoms may thus elevate the critical resolved shear stress (CRSS) required for dislocation nucleation and initial slip.

Upon entering the UTS and plastic flow stages, the dislocation evolution paths of the two systems diverge completely. In the cluster systems (Figure 8), deformation radiates outward from the central cluster as a core, where a large number of proliferating dislocation lines undergo intense piling up in front of the massive cluster. According to crystal plasticity theory, when the local pile-up stress exceeds a critical value, dislocation lines inevitably bend, thereby triggering the Orowan bypassing mechanism [32]. However, we note that the limited simulation cell size precludes direct observation of complete bypassing loops, and the observed dislocation configurations are suggestive of, rather than definitive proof of, this process. The continued slip of dislocations after interacting with the cluster interface may contribute to the gradual stress decline observed on the macroscopic curves. However, a deformation pattern that relies predominantly on a single interface may be prone to instability if that interface undergoes premature rupture.

In contrast, in the dispersed systems (Figure 7), the microstructure undergoes changes; the supercell interior becomes populated with dense, interleaved mobile dislocation networks and a large number of defect surfaces. These dislocation patterns strongly resemble forest dislocation hardening, in which dislocations on intersecting slip systems impede each other’s motion. We note, however, that DXA visualizations alone cannot definitively isolate forest hardening from other concurrent strengthening contributions that may also operate in these systems. In studies of complex solid solutions or high-entropy alloys, it has been proposed that potent dispersed solute atoms (such as Mo) can restrict the mean free path of dislocations, promoting frequent intersections and reactions among dislocations on different slip systems [33]. This global dislocation entanglement network provides a structural basis that likely accounts for the pronounced strain-hardening capability and the notably high tensile strength observed in dispersed systems such as Fe-Mo.

Of particular note is that in the late stages, where the structure is severely distorted or even near collapse, the W-doped system exhibits a unique “dislocation survival” characteristic, where a large number of dislocations remain active on the verge of failure. In the microscopic plasticity mechanisms of BCC metals, the non-planar core structure of screw dislocations often leads to sluggish glide. Specific alloying elements (such as W) may alter the nucleation and glide barriers of kink pairs on screw dislocations, which could facilitate cross-slip [14]. The sustained dislocation activity observed in the W-doped system near failure points toward a scenario where enhanced cross-slip contributes to the dissipation of localized deformation. We note, however, that unambiguous identification of individual cross-slip events from DXA analysis alone is challenging, and the present observations should be viewed as suggestive rather than conclusive. Such 3D slip reorganization may contribute to the dissipation of localized deformation work and could delay the expansion of micro-cracks, which offers a mechanistic rationale for the relatively high toughness observed in this system.

## 4. Conclusions

This study employed molecular dynamics simulations to systematically investigate the intrinsic effects of two spatial configurations (cluster vs. dispersion) of five transition metal elements (Co, Mo, Ni, Ti, and W) in a BCC iron matrix on micro-defect evolution and mechanical performance. Combining energy tracing, structural characterization, and defect kinetic analysis, the main conclusions are as follows.

(1) Spatial configuration influences macroscopic strength and toughness. Across all five solute elements, the dispersed configuration is consistently associated with higher ultimate tensile strength and greater ultimate strain compared with the corresponding cluster configuration. The Fe-Mo dispersed system reaches a UTS of 28.32 GPa (vs. 24.90 GPa for pure Fe under the same conditions), while the Fe-W dispersed system exhibits the largest ultimate tensile strain. The cluster configuration, by contrast, generally leads to reduced strength, a behavior driven by the stress concentration and premature dislocation pile-up at the interfaces.
(2) Thermodynamic stability and local atomic ordering correlate with strengthening in the dispersed configuration. The dispersed state consistently exhibits a more negative average potential energy per atom than the clustered state, indicating a more thermodynamically stable bonding network. RDF analysis reveals that the dispersed configuration is associated with a higher degree of local Fe–solute coordination. In the Fe-Mo system, the first-nearest-neighbor peak remains stable or increases at the UTS state—an observation indicative of stress-induced local atomic densification. In the Fe-W system, the observed disintegration of local coordination at large strains suggests that bond reorganization contributes to its higher toughness.
(3) Strain delocalization in the dispersed configuration avoids the interfacial weakness of clustered systems. In cluster configurations, extreme strain concentrates at the cluster–matrix interface. In the dispersed configuration, strain is distributed more uniformly throughout the matrix, supporting the hypothesis that global dispersion homogenizes deformation and delays crack initiation.
(4) Dislocation mechanisms transition from interface-localized to globally distributed. In cluster systems, dislocations nucleate preferentially at phase interfaces and their continued slip is suggestive of Orowan-type bypassing processes. In dispersed systems, dense, interleaved dislocation networks form throughout the matrix—patterns characteristic of forest dislocation hardening. The Fe-W dispersed system exhibits sustained dislocation activity up to large strains, implying that enhanced cross-slip plays a role in toughness.
(5) A “Mo-W composite dispersed” concept is proposed as a direction for further exploration. Based on the contrasting yet complementary mechanisms exhibited by Mo (local atomic ordering and extensive dislocation network formation) and W (sustained dislocation activity and higher toughness) in their respective single-solute dispersed configurations, a composite Mo-W dispersed design is proposed as a conceptual strategy. Rather than a validated engineering recommendation, this represents a hypothesis to be tested in future multi-component simulations and experiments. Experimental validation via APT and in situ TEM would be valuable for confirming the proposed mechanisms. Our future work will therefore be dedicated to multiscale modeling at slower strain rates, evaluating elevated-temperature stability, assessing cyclic corrosion fatigue, and exploring non-equilibrium synthesis pathways for realizing this Mo-W co-dispersed concept.

## Figures and Tables

**Table 1:** Ultimate tensile strength (UTS) and maximum uniform strain of the systems with different solute elements and doping configurations.

System/Element	Cluster Configuration		Dispersed Configuration	
	UTS(GPa)	Ultimate Strain	UTS(GPa)	Ultimate Strain
Pure Fe (Baseline)	24.90	0.0899	24.90	0.0899
Co	24.38	0.0904	23.62	0.0899
Mo	19.51	0.2423	28.32	0.3603
Ni	21.27	0.0781	21.93	0.2513
Ti	24.40	0.0939	24.50	0.0890
W	17.59	0.3846	20.90	0.4180
AVG (Exc. pure Fe)	21.43	0.1778	23.85	0.2417

Note: The maximum uniform strain is defined as the strain value corresponding to the UTS peak for each configuration.

## Data Availability

The original contributions presented in the study are included in the article, further inquiries can be directed to the corresponding author.
